# Which Is Right?

**Published:** 1870-10

**Authors:** 


### WHICH IS RIGHT?

The old woman said that the best way to preserve eggs the longest in good condition, was to put them away with the little end down, or the big end down, she did not remember which—but she was almost sure that it was one or the other.

Some who have taken calomel in large doses many times in their youth, and are now hale and hearty at three-score and more, with sound teeth, but few gray hairs, and never had a "rheumatic," think that calomel is good to take, and is one of the very best medicines ever used. Others, who never took a dose of it in their lives, say that it is a certain poison—rots the teeth, fills the joints with pains, makes a man scrofulous, and shortens his days, besides handing down his blighted constitution to his posterity. But now about

### FENCE POSTS.

Professor Daniels, of the University of Wisconsin, says there is no reason to believe that posts should be set in the ground

TOP DOWN, in order to make them last well. W. H. White writes to that favorite agricultural, *The Country Gentleman*, that twenty-three years ago he put in some fence posts, half top down and half in the position in which they grew; and that he observed no special difference, except that those which had the most sap were most decayed. The posts were sawed from red chestnut logs.

Then comes along R. C. Strong, Esq., from Louisa County, Iowa, through the well-edited columns of our old friend the *Prairie Farmer*, and says in a straightforward, intelligent way, that he made a gate post out of a thrifty white oak tree eighteen inches in diameter, in 1856. He split the timber in two pieces, put one in the ground top down, the other top up, or in the position in which it grew. The latter had rotted off, and fallen down at the end of ten years ; the other, which was put in top down, bid fair to last a dozen years longer.

Agricultural papers lose the confidence of their subscribers sometimes for want of definite specifications.

One kind of manure is good for one quality of soil and specific crop, but for another soil and a different crop it is worthless.

SETTING OUT FRUIT TREES.

Some years ago a great ado was made over a gardener who instructed his employer that the surest way to make his young trees thrive, was to put some stones in the bottom of the hole ; and it worked admirably. Another man might dig a hole a mile deep, and put in all the rocks on the top of Mount Washington, and every tree would die in a week. In a very wet soil, rocks would be a preventative against excessive moisture, for the rains would sink down between them, a kind of drain ; then again if the rocks were limestone, they would feed the tree, and the land there might have needed the lime element. There is nothing of greater importance in disease than, first, to be sure what the disease is, and that the remedy proposed is the same that cured in a similar case. Two heavily loaded donkeys came to a swollen stream in an over-heated and almost exhausted condition ; one plunged in and reached the opposite shore greatly refreshed, feeling that his load was not half so heavy as it was ; the other felt so much encouraged, that he plunged into the angry stream and sank to the bottom. He was loaded with wool, which with the absorbed waters pressed him down as he attempted to emerge from the other side of the river. The other was loaded with salt, which was melted and lightened by the water.

Sickness is often aggravated, and life itself is sometimes lost, from giving to a second man what cured the first, because the circumstances were not the same.

